# Remarkable genetic diversity of *Trypanosoma cruzi* and *Trypanosoma rangeli* in two localities of southern Ecuador identified via deep sequencing of mini-exon gene amplicons

**DOI:** 10.1186/s13071-020-04079-1

**Published:** 2020-05-14

**Authors:** Jalil Maiguashca Sánchez, Salem Oduro Beffi Sueto, Philipp Schwabl, Mario J. Grijalva, Martin S. Llewellyn, Jaime A. Costales

**Affiliations:** 1 Centro de Investigación para la Salud en América Latina, Escuela de Ciencias Biológicas, Facultad de Ciencias Exactas y Naturales, Pontificia Universidad Católica del Ecuador, Quito, Ecuador; 2 Institute of Biodiversity, Animal Health & Comparative Medicine, University of Glasgow, Glasgow, G128QQ UK; 3 Present Address: Institute for Biostatistics and Informatics in Medicine and Ageing Research, Rostock University Medical Center, 18057 Rostock, Germany; 4 Infectious and Tropical Disease Institute, Department of Biomedical Sciences, Heritage College of Osteopathic Medicine, Ohio University, Athens, OH 45701 USA

**Keywords:** Chagas disease, Mini-exon, *Trypanosoma cruzi*, *Trypanosoma rangeli*

## Abstract

**Background:**

*Trypanosoma cruzi*, the causative agent of Chagas disease, and *T. rangeli* are kinetoplastid parasites endemic to Latin America. Although closely related to *T. cruzi* and capable of infecting humans, *T. rangeli* is non-pathogenic. Both parasite species are transmitted by triatomine bugs, and the presence of *T. rangeli* constitutes a confounding factor in the study of Chagas disease prevalence and transmission dynamics. *Trypanosoma cruzi* possesses high molecular heterogeneity: seven discrete typing units (DTUs) are currently recognized. In Ecuador, *T. cruzi* TcI and *T. rangeli* KP1(-) predominate, while other genetic lineages are seldom reported.

**Methods:**

Infection by *T. cruzi* and/or *T. rangeli* in different developmental stages of triatomine bugs from two communities of southern Ecuador was evaluated via polymerase chain reaction product size polymorphism of kinetoplast minicircle sequences and the non-transcribed spacer region of the mini-exon gene (*n* = 48). Forty-three mini-exon amplicons were also deep sequenced to analyze single-nucleotide polymorphisms within single and mixed infections. Mini-exon products from ten monoclonal reference strains were included as controls.

**Results:**

*Trypanosoma cruzi* genetic richness and diversity was not significantly greater in adult vectors than in nymphal stages III and V. In contrast, instar V individuals showed significantly higher *T. rangeli* richness when compared with other developmental stages. Among infected triatomines, deep sequencing revealed one *T. rangeli* infection (3%), 8 *T. cruzi* infections (23.5%) and 25 *T. cruzi* + *T. rangeli* co-infections (73.5%), suggesting that *T. rangeli* prevalence has been largely underestimated in the region. Furthermore, deep sequencing detected TcIV sequences in nine samples; this DTU had not previously been reported in Loja Province.

**Conclusions:**

Our data indicate that deep sequencing allows for better parasite identification/typing than amplicon size analysis alone for mixed infections containing both *T. cruzi* and *T. rangeli*, or when multiple *T. cruzi* DTUs are present. Additionally, our analysis showed extensive overlap among the parasite populations present in the two studied localities (*c.*28 km apart), suggesting active parasite dispersal over the study area. Our results highlight the value of amplicon sequencing methodologies to clarify the population dynamics of kinetoplastid parasites in endemic regions and inform control campaigns in southern Ecuador.
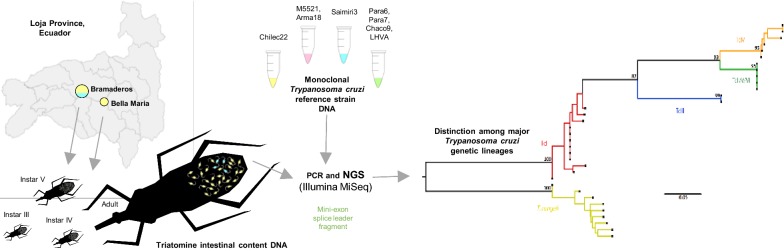

## Background

Chagas disease (CD) is caused by the kinetoplastid hemoflagellate parasite *Trypanosoma cruzi* and affects 6–7 million people worldwide [1]. This neglected disease is endemic to Latin America, where it poses risk of infection for > 65 million people and kills an estimated 50,000 every year [2]. Transmission in endemic countries primarily occurs via contact with the feces of an infected triatomine bug. However, other secondary mechanisms of transmission, such as blood transfusion, organ transplants, congenital transmission and laboratory accidents exist, and can cause infections in non-endemic regions where vectors are not present [3].

*Trypanosoma cruzi* has high molecular and phenotypic heterogeneity, and the existence of genetic lineages has been long recognized. Initially, three “zymodemes” (termed Z1, Z2 and Z3) were identified via multilocus enzyme electrophoresis [4, 5]. Subsequently, a wide variety of molecular markers have been developed and employed to demonstrate the existence of seven lineages or discrete typing units (DTUs), termed TcI-TcVI and Tcbat [6–12]. In Ecuador, TcI predominates in the central coast [13, 14] and southern highlands (Loja Province) [15]. Only two previous reports suggest the occurrence of parasites belonging to DTUs other than TcI, in triatomines and in patients of CD [16, 17].

In Ecuador, *Triatoma dimidiata*, *T. carrioni*, *Panstrongylus chinai*, *P. rufotuberculatus* and *Rhodnius ecuadoriensis* are epidemiologically relevant vector species [18, 19]. *Rhodnius ecuadoriensis* is widely distributed in the western lowlands and the southern provinces of the country, as well as in northern Peru [20], both in domestic and in peridomestic environments where contact with humans occurs [19, 21].

*Trypanosoma rangeli*, a related kinetoplastid parasite whose geographical distribution overlaps with that of *T. cruzi*, may also be found in the same triatomine vectors and mammalian hosts. Mixed infections with both species are often reported [18, 22, 23]. Despite being considered non-pathogenic to humans, *T. rangeli* is epidemiologically relevant since it may cause false-positive results in microscopical and serological tests used for diagnosis of *T. cruzi* infection [24, 25]. In contrast to that of *T. cruzi*, transmission of *T. rangeli* to vertebrate hosts occurs via the salivary route, although the possibility of transmission through infected feces has also been suggested [26, 27]. Upon ingestion from the circulation of vertebrate hosts, *T. rangeli* trypomastigotes accumulate in the digestive tract of triatomines, replicate, transform into epimastigotes, cross the intestinal epithelium into the hemocoel and migrate to the salivary glands. Infective metacyclic trypomastigotes are released with the saliva during a blood meal [28]. Little is known regarding *T. rangeli* in Ecuador. PCR minicircle amplification of > 3600 samples revealed that 10% of triatomines and mammals from Manabí and Loja provinces were infected with *T. rangeli*, while 1.25% presented *T. cruzi + T. rangeli* co-infections [29].

The kinetoplast DNA of trypanosomatid parasites is composed of maxicircles and minicircles, whose number varies between species. Minicircles possess a conserved 100–200 bp repetitive region, whose number of repeats also differs among species. In *T. cruzi*, there are four copies of the conserved sequence [30]. *Trypanosoma rangeli* may present one, two or four copies of the mini-repeat. These three different minicircle classes in *T. rangeli* are known respectively as KP1, KP2 and KP3 [31, 32]. Further analysis based on polymorphism of randomly amplified DNA (RAPD), sequencing of the small subunit of ribosomal RNA (*SSU* rRNA), the internal transcribed spacer of rDNA (ITS rDNA) and the intergenic region of the splice leader, has unveiled a more complex population structure for *T. rangeli*, with five groups defined as A, B, C, D and E, each of which shows a strong association with vector species and geographical distribution [27, 33, 34]. More recently, through the analysis of microsatellites and single-nucleotide polymorphisms (SNPs) of the splice leader gene, Sincero et al. [35] suggested the presence of three main groups in *T. rangeli*: (i) the Amazonian group, associated to *Rhodnius brethesi* and vertebrate hosts; (ii) the KP1(-) group, linked to the *R. pallescens* complex; and (iii) the KP1(+) group, related to the *R. robustus* complex.

Infection by *T. cruzi* in mammalian reservoirs and invertebrate vectors is frequently multiclonal, involving several intra-specific genotypes with genetically dissimilar profiles [36–39]. Multiclonal parasitic infections have been suggested to impact host immunity [40], disease transmission rate and population structure [41]. They can also mislead drug resistance evaluation, diagnostics and various other applications important to disease control [42].

In the genus *Trypanosoma*, mini-exon genes are present in several tandemly arranged copies and encode the splice leader, a 35-nucleotide sequence translocated to the 5’-end of every newly synthesized mRNA [43, 44] in a process referred to as discontinuous transcription [45]. *Trypanosoma cruzi* mini-exon genes include conserved, semi-conserved and highly variable regions, which have been used for phylogenetic analysis [46], discrimination between DTUs [47], population genetic inference [48], and more recently, in diversity analysis within naturally infected mammalian hosts [49]. Nonetheless, it is not clear how mini-exon sequence diversity relates to DTU identity, and restriction fragment length polymorphism (PCR-RFLP) assays targeting the mini-exon locus are not considered reliable for *T. cruzi* DTU detection and identification [50]. Given its variability, however, sequencing of the intergenic spacer has proven useful in the examination of population dynamics within a single DTU [50–55].

Next-generation sequencing (NGS) has helped provide insights into multiclonal *T. cruzi* infections in humans [56] and to assess relationships between gut microbiota, parasite diversity, and vertebrate feeding sources of triatomine bugs [57]. In the present study, we aimed to evaluate the molecular diversity of *T. cruzi* and *T. rangeli* in two localities of southern Ecuador via analysis of the mini-exon gene. We analyzed 48 intestinal DNA extracts from triatomines collected in Loja Province as well as DNA from ten cloned isolates of reference strain DTUs I, III, IV, V and VI in order to identify relationships between mini-exon diversity and DTU identity. Mini-exon amplicon sequence reads generated by NGS were sorted into haplotype clusters to measure genetic richness, diversity, variability and substructure in the landscape. Moreover, the dataset included samples isolated from different developmental stages of the vector to evaluate a potential correlation with mini-exon diversity. We also compared genotyping results from NGS and PCR product size polymorphism analysis in terms of sensitivity for discrimination between *T. cruzi* and *T. rangeli* parasites.

## Methods

### Sample panel and PCR typing

Archived DNA samples collected between years 2009–2013 by the Center for Research on Health in Latin America (CISeAL) were employed in the study. Details of entomological searches, intestinal content DNA extractions and corresponding approved protocols and collection permits have been reported elsewhere [19, 23]. Samples selected for the study had been previously genotyped by PCR amplification using primers 121 and 122, which anneal to highly conserved regions of the kinetoplast minicircle [58] and are useful to distinguish between *T. cruzi* (330 bp fragment size expected) and *T. rangeli* (380 bp). All samples appeared to be infected exclusively with *T. cruzi* based on visualization of 121/122 amplicons (data not shown).

**Fig. 1:**
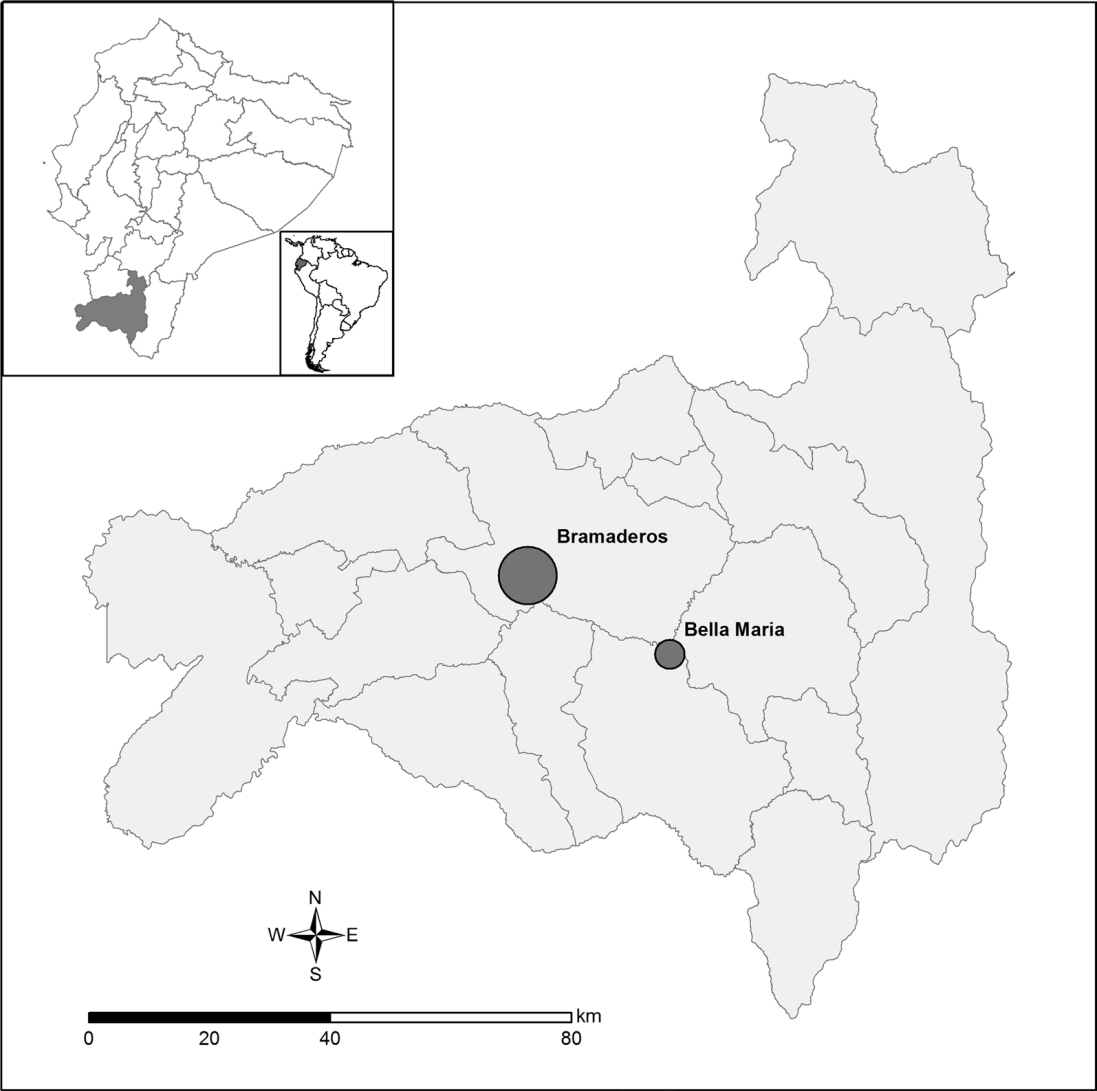
Study area. The two studied communities within Loja Province in southern Ecuador are depicted. They are separated by approximately 28.3 km. Circle diameter is proportional to sample size in each locality. Map generated with ArcGIS software, version 10.5, based on data freely available from the Military Geographic Institute of Ecuador (IGM, http://www.geoportaligm.gob.ec/portal/). Community location was obtained via GPS measurement

Forty-eight samples from two rural communities from southern Ecuador (Loja Province) were included in the sample panel: 31 from Bramaderos (4°4′47″S, 79°49′28″W); and 17 from Bellamaría (4°12′41″S, 79°36′23″W) (Fig. 1). Hypothesizing that infection multiclonality might accumulate with blood meal number, we characterized the molecular diversity/multiclonality of the mini-exon gene of the parasites across vector developmental stages, including samples obtained from 20 adult, 14 instar V, 3 instar IV and 11 instar III triatomine bugs.

The mini-exon gene is a multicopy target. DNA from 10 biologically cloned reference strains was therefore included in the analysis to evaluate whether intra-clonal sequence diversity generated from the amplicons via NGS recapitulated the DTU to which the clones had been previously assigned via classical methods [50].

### Multiplex PCR

PCR amplification of the non-transcribed spacer region of the mini-exon gene was performed employing five primers in a multiplex reaction to discriminate between zymodemes I, II, III and *T. rangeli*, as previously described [47]. In the current nomenclature zymodeme I is equivalent to DTU TcI, zymodeme II comprises DTUs II, V and VI, while zymodeme III corresponds to DTUs III and IV. The amplified products were electrophoresed in 2% agarose gels and visualized with SYBR green under UV light. Samples co-infected with *T. cruzi* and *T. rangeli* displayed a two-band pattern. In such cases, gel excision and purification were performed for each fragment. Subsequently, amplicons were sequenced by NGS technology as detailed below.

### Next-generation sequencing and bioinformatic analysis

Paired-end sequence reads were generated on the Illumina MiSeq platform (600-cycle Reagent Kit v3; Illumina, San Diego, USA) following a custom protocol [59]. Paired-end sequence reads were processed with Cutadapt v1.12 for adapter removal and Sickle v1.33 for quality trimming (-q 27) [60]. Trimmed forward and reverse reads were overlapped using default settings in Pandaseq v2.7 [61]. Eleven of 56 sample barcodes used during multiplexed sequencing in this study were also used for a separate study on *18S* gene amplicons. These *18S* amplicons were sequenced in the same MiSeq flow cell in order to save sequencing costs. We discarded all sequences containing *18S* k-mer matches after the Pandaseq overlap step. Sequences with > 98% sequence similarity were then clustered to consensus haplotypes based on the UPARSE-OTU algorithm implemented by the ‘cluster_otus’ command in USEARCH v8.1 [62]. Chimeric sequences were discarded using the UCHIME algorithm and clusters represented by < 200 reads were discarded to avoid artefactual SNPs. Samples for which the ‘usearch_global’ command assigned < 20,000 reads to the remaining set of consensus sequences were also discarded based on rarefaction curves computed from cluster size annotations using ‘fasta_rarify’. Finally, parasite species and DTUs were determined for each haplotype by searching the complete nucleotide collection (nr/nt) with the Basic Local Alignment Search Tool (BLAST) at the National Center for Biotechnology Information (NCBI). All sequences with ≥ 90% similarity to trypanosomatid mini-exon sequences were kept for further analysis. (Additional file 1: Table S1 shows raw data for cloned reference strains and Ecuadorian samples).

### Richness and diversity analysis

Richness was defined as the total number of haplotypes found in each sample. Shannonʼs index was defined as -Ʃ(Piln(Pi)) [63]. As mentioned previously, samples with mixed infections showed a two-band pattern in agarose gels. In these cases, gel excision rendered two sequencing templates and the information obtained was merged (addition of reads from each template) (Additional file 2: Table S2). For each sample, richness and diversity were calculated for both parasite species (*T. cruzi* and *T. rangeli*) independently and combined. Data corresponding to samples TBM2823, TBR1487 and TBR1489 were excluded from this analysis because they are the only representatives of instar IV. TBR1446 and TBR1503 were also excluded to avoid bias because the sequence reads from these samples has been merged across four sequencing reactions.

### Statistical analysis

An analysis of variance (ANOVA) was performed when data showed normality and homoscedasticity. When data violated normality, a Kruskal-Wallis test was employed, followed by a Bonferroni-adjusted Dunn’s *post-hoc* test. A *P*-value of ≤ 0.05 was considered significant.

## Results

### Concordance between mini-exon sequence diversity and DTU identity

**Fig. 2:**
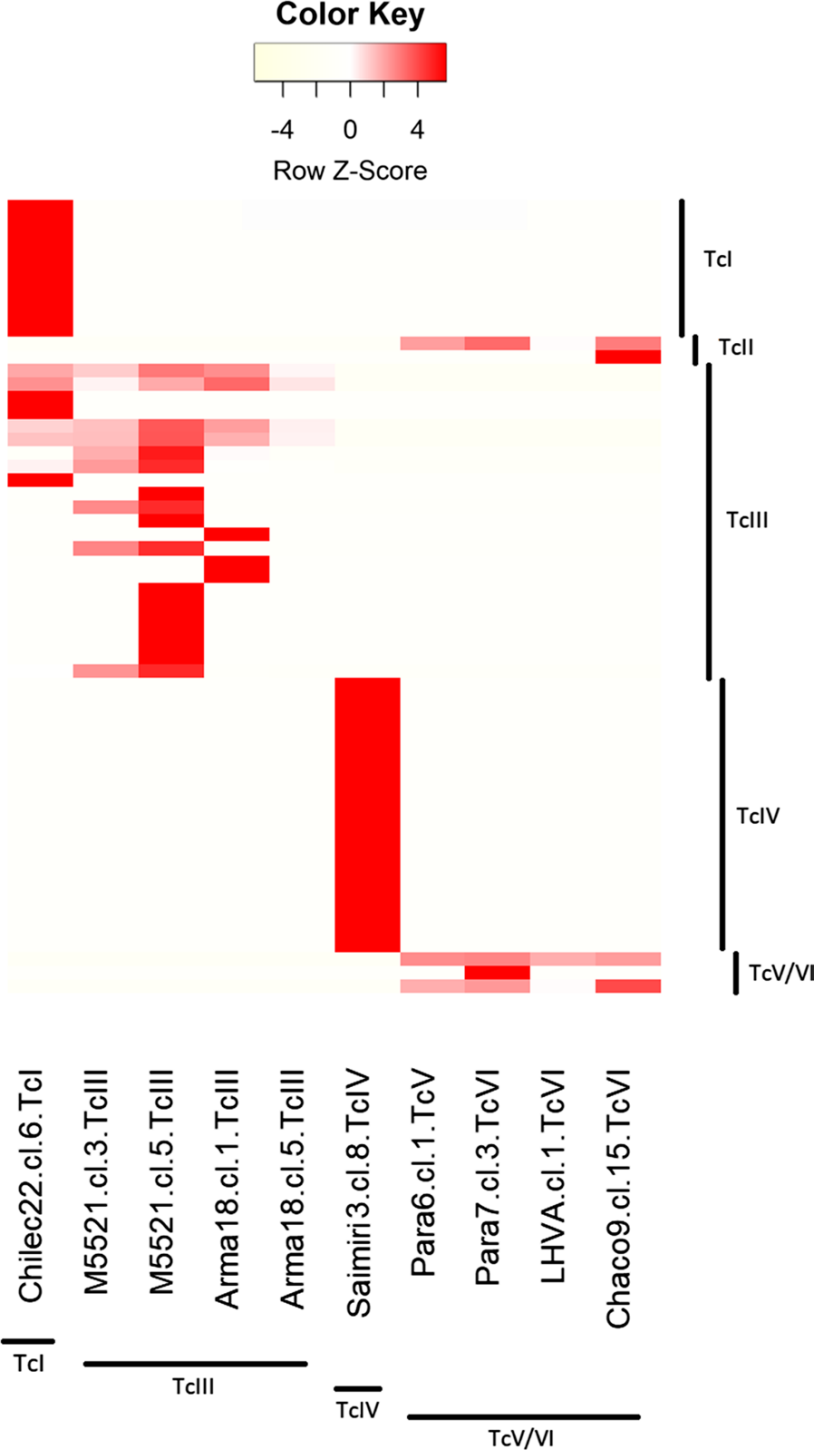
Distinctive sequence types derived from monoclonal samples. Monoclonal samples from reference strains are displayed on the x-axis. The y-axis includes distinctive sequence types identified in this study that showed association with reference strains (haplotypes seen almost exclusively within a single monoclonal sample as representative of a DTU). Color intensity is proportional to the number of reads, here standardized for each row (sequence/haplotype) and displayed as a *z*-score

The monoclonal reference strains included in the study were Chilec22 cl.6 (TcI), M5521 cl.3 and cl.5 (TcIII), Arma18 cl.1 and cl.5 (TcIII), Saimiri3 cl.8 (TcIV), Para6 cl.1 (TcV), Para7 cl.3 (TcVI), Chaco9 cl.15 (TcVI) and LHVA cl.1 (TcVI) [10, 50, 64]. The mini-exon gene sequence was analyzed for each of these monoclonal reference strains, revealing a predominant haplotype (~98% of sequences) with one or few different sequences in most samples (Fig. 2).

### Genotyping by multiplex PCR and NGS

**Fig. 3:**
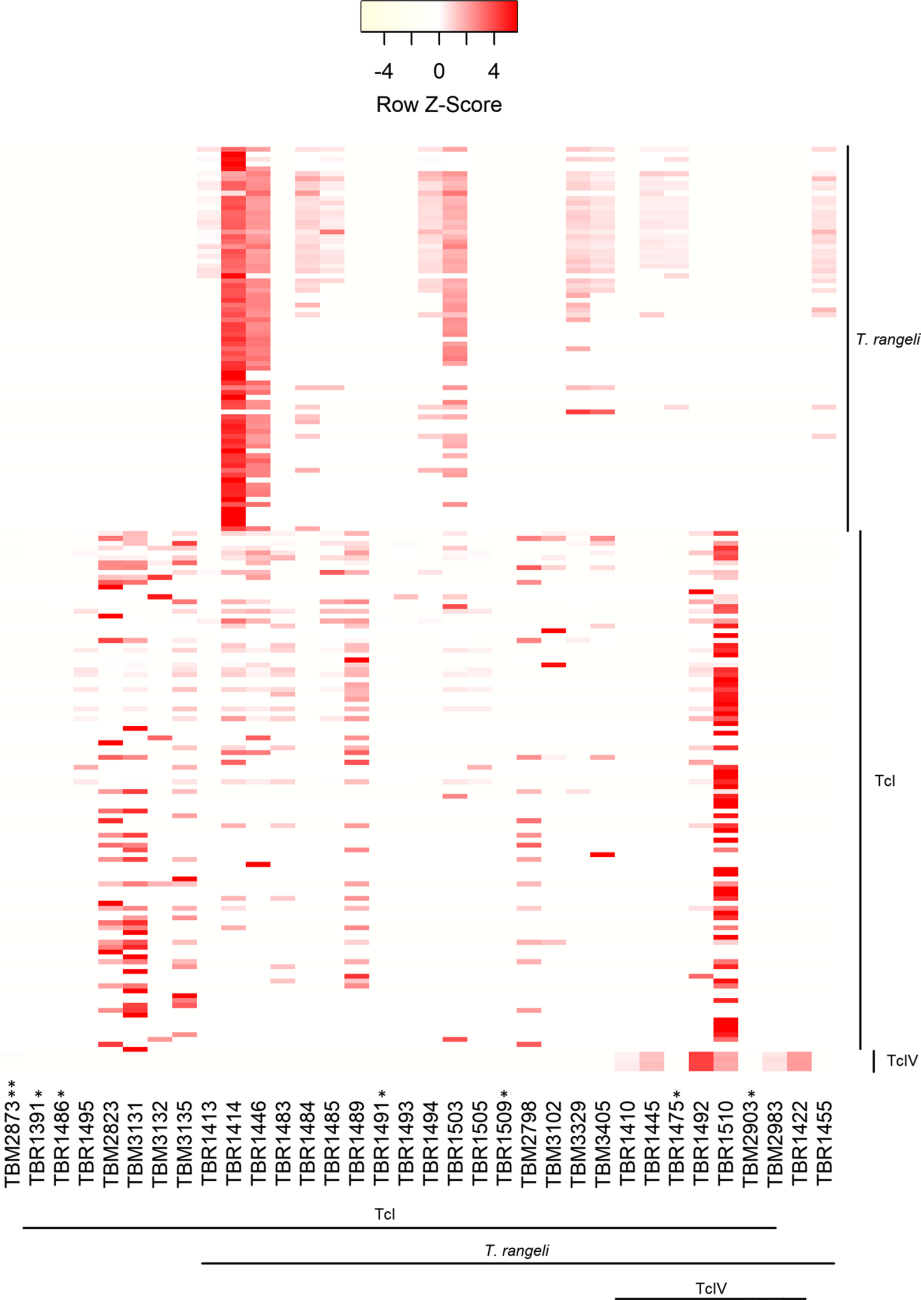
Sequence types associated with *T. cruzi* and/or *T. rangeli* amplified from intestinal content of Ecuadorian triatomines. The y-axis shows the sequence types and their assigned DTU according to their similarity with GenBank reference sequences (> 98%). Samples are shown in the x-axis, indicating their assigned species and DTU. Color intensity is proportional to the number of reads, here standardized for each row (sequence/haplotype) and displayed as a *z*-score. * Indicates that the number of reads for these samples is too low to be visualized in the *z*-score adjusted heatmap. Among these, ** TBM2873 is the only sample where a TcI/TcIV co-infection was detected

Among the 48 samples analyzed by multiplex PCR, agarose electrophoresis patterns indicated that 37.5% (*n* = 18) of the samples were co-infected with *T. cruzi* and *T. rangeli*, while 52.1% (*n* = 25) presented infection exclusively with *T. cruzi* and 6.25% (*n* = 3) were infected only with *T. rangeli*. Samples TBM2873 and TBM2983 showed faint bands in agarose gels and no clear pattern was obtained. Meanwhile, NGS was performed only on 43 samples from the Ecuadorian dataset; 5 were not sequenced due to technical issues. An additional 9 samples were excluded from further analysis because they did not reach the 20,000 reads threshold, indicating insufficient sequencing depth. Among the 34 samples for which NGS was successfully performed, taxonomic assignment in GenBank identified sequence types associated with both *T. cruzi* and *T. rangeli* in 25, while 8 were deemed to contain exclusively *T. cruzi*-like sequences, and only one had exclusively *T. rangeli*-like sequences (TBR1455). Remarkably, sequence types matching DTU TcIV were found in samples TBR1410, TBR1422, TBR1445, TBR1475, TBR1492, TBR1510, TBM2873, TBM2903 and TBM2983 (Table 1, Fig. 3).

**Table 1:** Samples employed in the study and genotyping results

Vector species	Sample code	Community	Instar/sex^a^	Multiplex PCR	NGS
*P. chinai*	TBM2798	Bellamaria	V	TcI	*T. rangeli/*TcI
*P. chinai*	TBM2823	Bellamaria	IV	TcI	TcI
*P. chinai*	TBM2824	Bellamaria	III	TcI	Reads < 20000
*R. ecuadoriensis*	TBM2841	Bellamaria	Male	*T. rangeli/*TcI	na
*R. ecuadoriensis*	TBM2873	Bellamaria	Female	Faint bands	TcI/TcIV
*R. ecuadoriensis*	TBM2903	Bellamaria	III	TcI	*T. rangeli*/TcI/TcIV
*R. ecuadoriensis*	TBM2972	Bellamaria	Female	*T. rangeli*	Reads < 20000
*R. ecuadoriensis*	TBM2982	Bellamaria	V	TcI	Reads < 20000
*R. ecuadoriensis*	TBM2983	Bellamaria	V	Faint bands	*T. rangeli*/TcI/TcIV
*R. ecuadoriensis*	TBM3102	Bellamaria	Female	TcI	*T. rangeli*/TcI
*R. ecuadoriensis*	TBM3131	Bellamaria	III	TcI	TcI
*R. ecuadoriensis*	TBM3132	Bellamaria	Female	TcI	TcI
*R. ecuadoriensis*	TBM3135	Bellamaria	Female	TcI	TcI
*R. ecuadoriensis*	TBM3329	Bellamaria	V	*T. rangeli/*TcI	*T. rangeli/*TcI
*R. ecuadoriensis*	TBM3376	Bellamaria	III	*T. rangeli/*TcI	na
*R. ecuadoriensis*	TBM3377	Bellamaria	III	TcI	Reads < 20000
*R. ecuadoriensis*	TBM3405	Bellamaria	Male	*T. rangeli/*TcI	*T. rangeli/*TcI
*R. ecuadoriensis*	TBR1379	Bramaderos	III	TcI	Reads < 20000
*R. ecuadoriensis*	TBR1391	Bramaderos	Male	TcI	TcI
*R. ecuadoriensis*	TBR1409	Bramaderos	Male	*T. rangeli/*TcI	na
*R. ecuadoriensis*	TBR1410	Bramaderos	Male	TcI	*T. rangeli*/TcI/TcIV
*R. ecuadoriensis*	TBR1413	Bramaderos	V	*T. rangeli/*TcI	*T. rangeli/*TcI
*R. ecuadoriensis*	TBR1414	Bramaderos	V	*T. rangeli/*TcI	*T. rangeli/*TcI
*R. ecuadoriensis*	TBR1422	Bramaderos	III	*T. rangeli*	*T. rangeli/*TcIV
*R. ecuadoriensis*	TBR1432	Bramaderos	Female	*T. rangeli/*TcI	Reads < 20000
*R. ecuadoriensis*	TBR1445	Bramaderos	III	*T. rangeli/*TcI	*T. rangeli*/TcI/TcIV
*R. ecuadoriensis*	TBR1446	Bramaderos	V	*T. rangeli/*TcI	*T. rangeli/*TcI
*R. ecuadoriensis*	TBR1455	Bramaderos	V	*T. rangeli/*TcI	*T. rangeli*
*R. ecuadoriensis*	TBR1470	Bramaderos	Male	TcI	na
*R. ecuadoriensis*	TBR1475	Bramaderos	Male	*T. rangeli/*TcI	*T. rangeli*/TcI/TcIV
*R. ecuadoriensis*	TBR1480	Bramaderos	V	*T. rangeli*	Reads < 20000
*R. ecuadoriensis*	TBR1483	Bramaderos	Female	TcI	*T. rangeli*/TcI
*R. ecuadoriensis*	TBR1484	Bramaderos	III	*T. rangeli/*TcI	*T. rangeli/*TcI
*R. ecuadoriensis*	TBR1485	Bramaderos	III	*T. rangeli/*TcI	*T. rangeli/*TcI
*R. ecuadoriensis*	TBR1486	Bramaderos	III	TcI	TcI
*R. ecuadoriensis*	TBR1487	Bramaderos	IV	TcI	na
*R. ecuadoriensis*	TBR1489	Bramaderos	IV	TcI	*T. rangeli*/TcI
*R. ecuadoriensis*	TBR1491	Bramaderos	V	TcI	*T. rangeli/*TcI
*R. ecuadoriensis*	TBR1492	Bramaderos	V	*T. rangeli/*TcI	*T. rangeli/*TcI*/*TcIV
*R. ecuadoriensis*	TBR1493	Bramaderos	Male	TcI	*T. rangeli/*TcI
*R. ecuadoriensis*	TBR1494	Bramaderos	Male	*T. rangeli/*TcI	*T. rangeli/*TcI
*R. ecuadoriensis*	TBR1495	Bramaderos	Male	TcI	TcI
*R. ecuadoriensis*	TBR1502	Bramaderos	V	TcI	Reads < 20000
*R. ecuadoriensis*	TBR1503	Bramaderos	V	*T. rangeli/*TcI	*T. rangeli*/TcI
*R. ecuadoriensis*	TBR1505	Bramaderos	Female	*T. rangeli/*TcI	*T. rangeli/*TcI
*R. ecuadoriensis*	TBR1508	Bramaderos	V	TcI	Reads < 20000
*R. ecuadoriensis*	TBR1509	Bramaderos	Male	TcI	*T. rangeli*/TcI
*R. ecuadoriensis*	TBR1510	Bramaderos	Female	TcI	*T. rangeli/*TcI*/*TcIV

^a^Developmental stage from the triatomine vectors. Roman numerals correspond to nymphal instars. Sex is indicated for adult triatomines

*Abbreviation*: na, not available

**Fig. 4:**
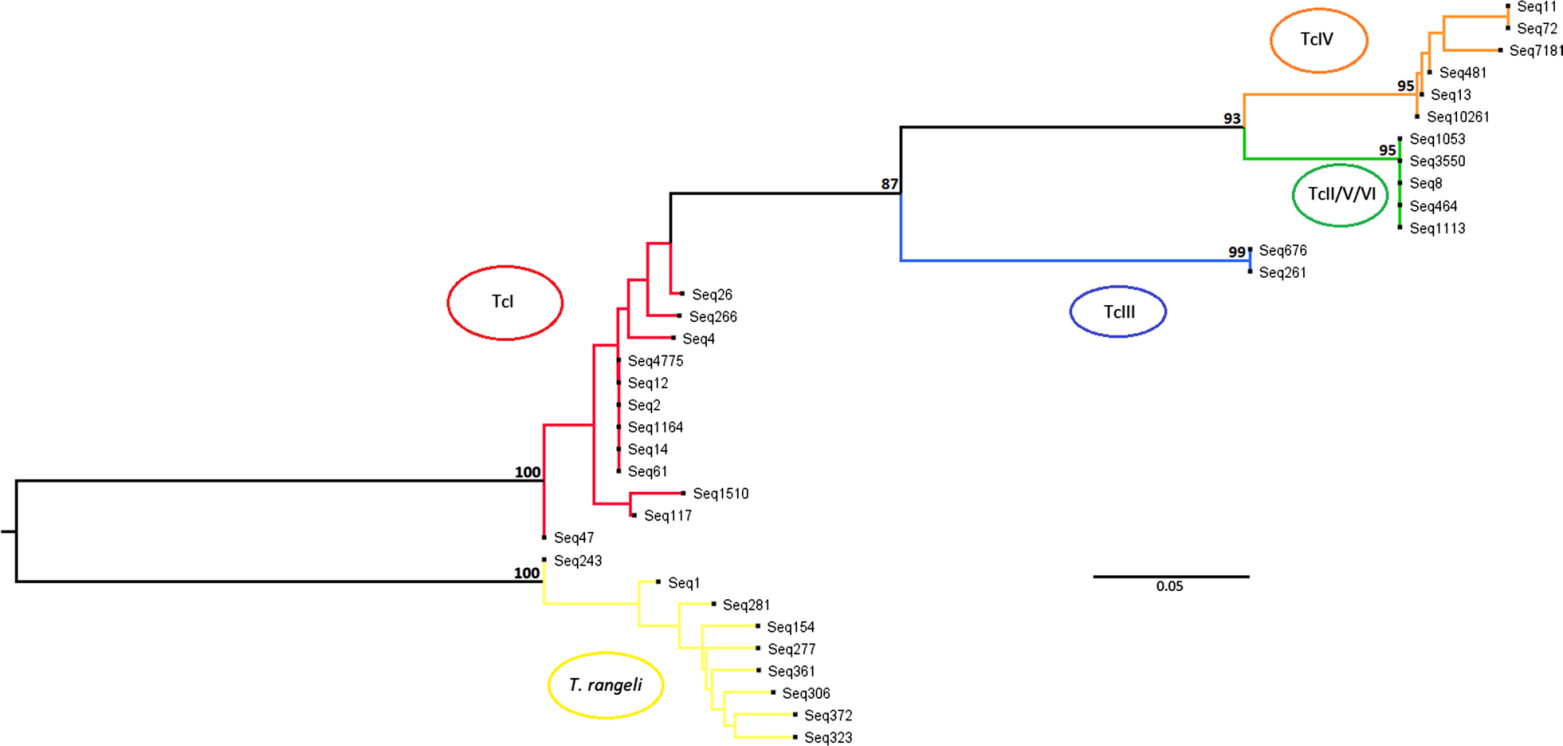
NJ analysis of selected mini-exon sequence types derived from Ecuadorian samples and monoclonal reference strains. Rooted tree (user selection) generated in MEGA7. Alignment performed by Muscle and manually edited. All haplotypes showing > 10,000 reads were included. Other haplotypes with < 10,000 reads were included to ensure all DTUs were represented

Mini-exon sequence types distinctively associated with monoclonal strains TcI, TcIII, TcIV and TcV/VI were used for the construction of a neighbor-joining (NJ) tree alongside selected sequence types derived from Ecuadorian strains and *T. rangeli*. Haplotypes with more than 10,000 reads were selected, although key sequences associated with a specific DTU and Ecuadorian TcIV representatives were included in the alignment (Additional file 3: Alignment S1). The tree topology shows five robustly supported clusters, one corresponding to *T. rangeli*, separated from four corresponding to *T. cruzi*. The TcI-like sequences cluster together in one branch, separated from TcIV-like, TcIII-like and TcII/V/VI-like sequences (Fig. 4).

**Fig. 5:**
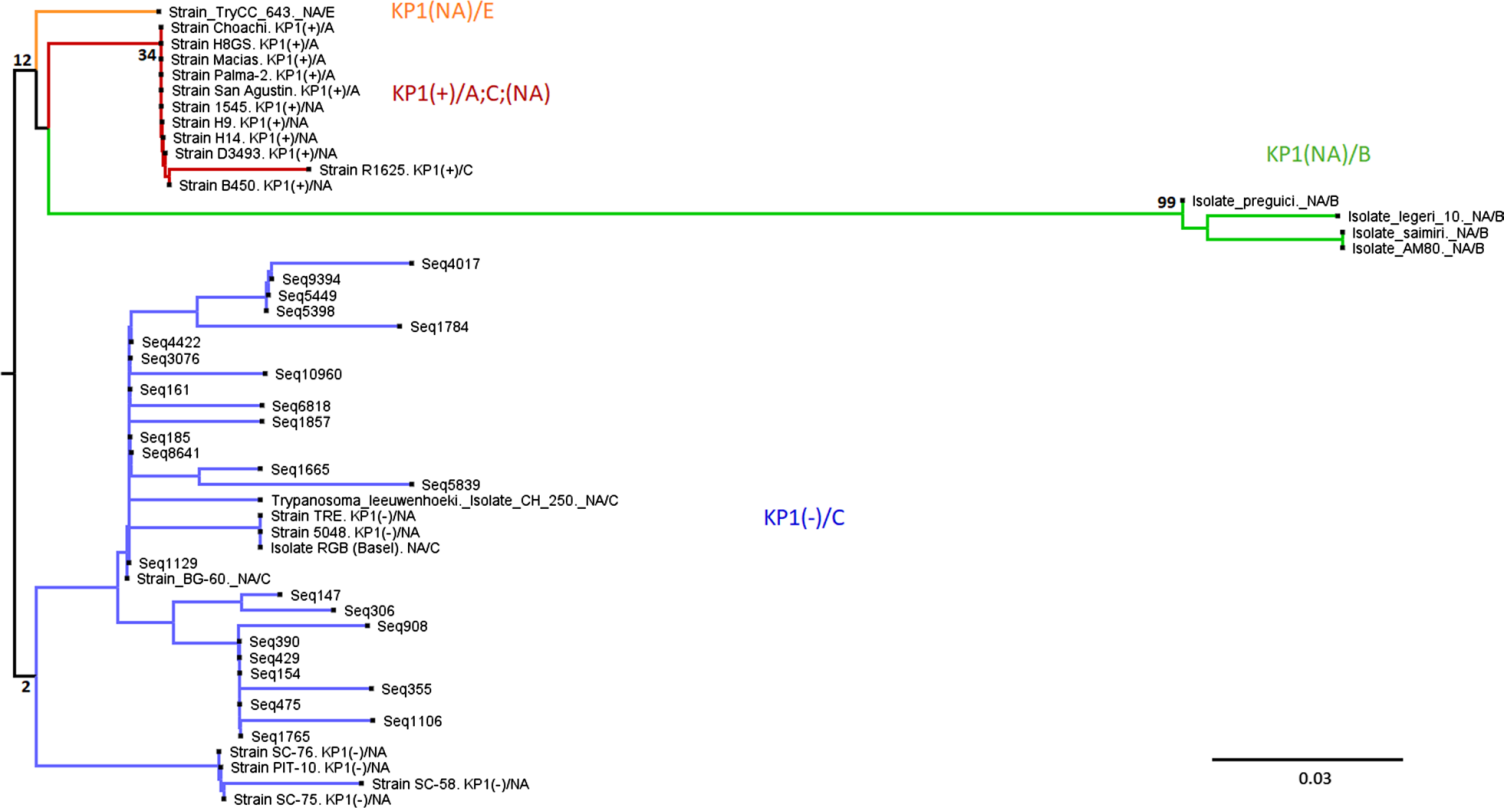
NJ analysis of selected *T. rangeli* haplotypes derived from Ecuadorian samples. Rooted tree (user selection) generated in MEGA7. Alignment performed by Muscle and manually edited. Reference sequences available at the NCBI database are included (Additional file 4: Alignment S2 shows the alignment used for tree generation and Additional file 5: Table S3 shows the sequences retrieved from the NCBI database)

NJ analysis including Ecuadorian *T. rangeli* haplotypes yielded a rather uninformative clustering with several polytomies. Therefore, only selected *T. rangeli* haplotypes were included along with available sequence data retrieved from NCBI (Fig. 5, Additional file 4: Alignment S2, Additional file 5: Table S3). The Ecuadorian haplotypes clustered with KP1(-)/C reference sequences. Although bootstrap values are low for most branches, KP1(+), KP1(−) and groups A, B, C and E [34] are also evident in the tree.

### Parasite richness and diversity

Chao richness and Shannonʼs diversity indices were calculated for each parasite species within each sample. No significant differences in richness or diversity were found between samples from the two studied localities (Bramaderos and Bellamaria) for either parasite species. Analyses were performed merging both male and female adults and with adults from each sex taken independently. In both cases, we found significant differences for *T. rangeli* richness across developmental stages (Kruskal-Wallis H-test: *χ*^2^ = 8.4478, *df* = 3, *P =* 0.0376 for male/female independent analysis, and Kruskal-Wallis H-test: *χ*^2^ = 6.8417, *df* = 2, *P* = 0.0327 for the merged group). The Bonferroni-adjusted Dunn’s *post-hoc* test showed significant differences between instar V nymphs and adult females (*P* = 0.0124) and among instar V nymphs and the merged group of adult females and males (*P* = 0.0138). In both cases, instar V showed significantly higher richness than the remaining developmental stages.

## Discussion

### Comparison of parasite identification/genotyping methods

We compared the results from three different types of molecular analysis for 48 intestinal content DNA samples from triatomine bugs collected in two rural communities of Loja Province, southern Ecuador. All samples selected for the study had been previously characterized as infected with *T. cruzi* based on PCR product size polymorphism of kinetoplast minicircle sequences, with no evidence of co-infection with the sympatric sister species *T. rangeli*. The sample set was subsequently analyzed by two additional molecular techniques; first by PCR product size polymorphism analysis of the non-transcribed spacer region of the mini-exon gene, then by deep sequencing of these mini-exon amplicons. Incongruences were detected among the results obtained by the three methods, and our results suggest that simple amplicon visualization of kinetoplast minicircle sequences may not suffice for detection of *T. cruzi + T. rangeli* co-infections.

The PCR-based method for mini-exon gene analysis without sequencing [47] uncovered 18 instances of *T. cruzi + T. rangeli* co-infections in our sample panel, which the kinetoplast minicircle PCR [58] did not identify. Mixed infections of *T. cruzi* and *T. rangeli* in vector and mammalian hosts are frequent [18, 23, 65, 66] and several groups have reported that minicircle kinetoplast PCR product visualization techniques are inadequate to identify them [66–69]. Previous studies have applied this same method in southern Ecuador and reported ~10% prevalence of *T. rangeli* infection in sampled triatomines and mammals and just ~1.25% in co-infection with *T. cruzi* [29]. In stark contrast, NGS revealed *T. rangeli*-like sequences in 26 out of 34 (76.47%) infected samples characterized in our study.

### *Trypanosoma rangeli and T. cruzi* genetic diversity

Our sample panel was heavily biased toward *R. ecuadoriensis* (45 out of 48 DNA samples were isolated from this vector species), one of the two most important vectors of CD in the study region and in Ecuador at large [70]. A close association between *T. rangeli* and *Rhodnius* spp. has been established in the past, where 12 out of 15 species of the genus have shown vectorial capacity [26]. Our results suggest that previous reports regarding *T. rangeli* infection in *R. ecuadoriensis* may underestimate the presence of this parasite in southern Ecuador, where a wider distribution of *T. rangeli* in Loja, and perhaps the rest of the country, may have been overlooked. Furthermore, although only a ~100-bp fragment was analyzed, a wide *T. rangeli* genetic diversity was encountered (79 haplotypes in 26 triatomines). Consistent with previous reports, where 99% of Ecuadorian *T. rangeli* were classified as KP1(−), and 80% as lineage C [29], NJ analysis clustered all Ecuadorian sample haplotypes with KP1(-)/C reference sequences. Low bootstrap values in this analysis may arise from the small size (~100 bp) of the *T. rangeli* amplicon and the limited number of informative SNPs found within the sequence (some haplotypes differ by only one SNP). Notably, we did not identify any KP1(+) haplotypes, although previous reports indicate KP1(+) and (−) lineages are sympatric and may be found in the intestinal contents of *Rhodnius* spp. [71].

Co-infection with *T. cruzi* and *T. rangeli* occurred in 73.5% of analyzed triatomines. Despite earlier reports suggesting *T. rangeli* infection is pathogenic to triatomines, more recent studies point to the need of more solid evidence before generalizing this claim [72]. Indeed, recent reports suggest co-infection increases *R. prolixus* survival, reproduction and fitness, which in turn would favor transmission of both parasite species [73]. The interplay of the infection with these two trypanosomatids in *R. ecuadoriensis* and its possible impact over the epidemiology of Chagas disease in the south of Ecuador warrants further investigation.

Sequencing of the non-transcribed spacer of the mini-exon gene has proven useful in phylogenetic analysis and has provided valuable insights for DTU discrimination while analyzing *T. cruzi* intra-strain variability [10, 48, 51, 55]. Recently, deep sequencing of the non-transcribed spacer has revealed high levels of *T. cruzi* diversity in the intestinal content of *T. dimidiata* [57]. Similarly, Herrera et al. [49] recently employed NGS sequencing of the mini-exon gene to analyze the genetic diversity of *T. cruzi* naturally infecting a group of captive non-human primates in the southern USA. In our study, 14.5 *T. cruzi* and 14.4 *T. rangeli* haplotypes were detected on average per triatomine. However, we do note from our analysis of monoclonal reference samples that mini-exon sequences may not accurately resolve *T. cruzi* phylogenetic diversity, thus reflecting to an extent the lack of reliability of this locus in identifying DTUs. From monoclonal strain haplotypes, our data showed that some low abundance sequence types are shared by almost all DTUs, with important implications for those using the mini-exon splice leader locus to define intra-host DTU diversity (Additional file 1: Table S1). Therefore, it is also difficult to say with confidence that the high levels of *T. cruzi* diversity found in our samples result from multiclonal infections, given that multiple divergent copies are present per genome. Nonetheless, a subset of high abundance sequence types could be associated with different DTUs and it is upon these that we base our discrimination between TcI and TcIV in this study.

TcI is the predominant DTU in Ecuador [13–15, 23]. As expected, TcI-like haplotypes were widely represented in the deep sequencing analysis of the mini-exon gene performed for the Ecuadorian samples. Notably, we identified sequences matching DTU TcIV in nine of the Ecuadorian samples. Two TcIV-like haplotypes displayed a 99% match with an NCBI entry originated from a TcIV isolate. The other two TcIV-like haplotypes found also showed high homology (97% and 98%). Additionally, NJ analysis clustered the Ecuadorian TcIV haplotypes with discriminatory TcIV sequences available from monoclonal strains with known DTU identity. Strong bootstrap values support this finding; therefore, we are confident of the identification of TcIV haplotypes in our sample panel. To the best of our knowledge, this constitutes the first report of TcIV in Loja Province and is a testament to the power of amplicon sequencing to uncover ‘hidden’ *T. cruzi* diversity. Only two previous reports of DTUs different from TcI in Ecuador exist. One of them, from the early 2000s, employed multilocus enzyme electrophoresis to identify zymodemes II and III [16]. The second report provided serological evidence using a TcII/V/VI-specific epitope [17]. Our findings expand the distribution of DTU TcIV to the southernmost province of Ecuador, where its presence had not previously been reported.

No major differences were found between the studied localities in terms of parasite genetic richness or diversity. Both communities are only 28.3 km apart and infected livestock, small mammals, passive transportation of triatomines or human movement may cause active parasite dispersal, homogenizing the parasite populations. In addition, no significant differences in *T. cruzi* richness or diversity were detected across vector developmental stages. The hostile environment in the vector’s anterior midgut has been previously suggested to create a bottleneck [74], which could reduce the diversity of *T. cruzi*. In contrast, the significantly higher *T. rangeli* richness found in instar V compared to adult triatomines is consistent with the notion of this parasite being pathogenic for triatomines [75, 76], which could affect their development into adulthood.

Dumonteil et al. [57] reported evidence of *T. cruzi* clone accumulation in *T. dimidiata* as a result of subsequent feeding events by this vector. The data do not support the occurrence of such accumulation in our sample set, which may be linked to the presence of multiple copies, and variants, of mini-exon sequences within clones, which obscure the signal of accumulating clonal diversity over multiple infected blood meals.

## Conclusions

Our results indicate that techniques based solely on PCR amplification of minicircle or mini-exon sequences are not optimal for detection and characterization of mixed infections with kinetoplastid parasites in triatomines. Employing deep sequencing of the non-transcribed spacer region of the mini-exon gene, we have encountered 73.5% (considering the 34 samples with NGS results) of studied triatomines to be co-infected with *T. cruzi* and *T. rangeli*, a much higher prevalence for *T. rangeli* infection than previously reported for vectors in the study region. Haplotypes of *T. rangeli* found in Ecuadorian samples were characterized as KP1(-)/C, in congruence with previous reports. Additionally, we provide the first report for the presence of DTU TcIV in Loja Province. In conclusion, we demonstrate the power of NGS to scrutinize parasite populations with high resolution. However, we do note that the mini-exon marker must be approached with caution, and further deep sequencing analyses targeted at additional markers, potentially conserved house-keeping genes [77], are required for confirmation. Nonetheless, the information presented in this report is of value in the characterization of the dynamics of *T. cruzi* transmission in southern Ecuador and must be considered in disease control efforts in the region.

## Supplementary information

**Additional file 1: Table S1.** Raw data for monoclones and Ecuadorian samples. Haplotype ID, identity and number of reads are included.

**Additional file 2: Table S2.** Correspondence between FLDs* (Illumina sequencer output) and samples belonging to intestinal contents.

**Additional file 3: Alignment S1.** Sequences employed for the phylogenetic tree displayed in Fig. 4.

**Additional file 4: Alignment S2.** Sequences employed for the phylogenetic tree displayed in Fig. 5.

**Additional file 5: Table S3.** Accession numbers of downloaded sequences from NCBI database used in the generation of the NJ tree for *T. rangeli*. The retrieved sequences were trimmed to match with the amplicons generated with Ecuadorian samples. The discarded portions of the sequences are shown. Lineage (A, B, C, D and E) and KP1(+/−) information was included when available. Geographical origin of the samples is also in the file.

## Data Availability

Data supporting the conclusions of this article are included within the article and its additional files. The datasets generated during this study are available at the sequence read archive (SRA) database, bioproject PRJNA596271, https://www.ncbi.nlm.nih.gov/sra/PRJNA596271. The alignment used for Fig. 4 is included in Additional file 3: Alignment S1, and the alignment used for Fig. 5 is included in Additional file 4: Alignment S2.

## Abbreviations

CD: Chagas disease
DTU: discrete typing unit
TcI: *Trypanosoma cruzi* I
PCR: polymerase chain reaction
RAPD: random amplification of polymorphic DNA
*SSU* rRNA: small subunit of ribosomal RNA
ITS rDNA: internal transcribed spacer of rDNA
SNP: single-nucleotide polymorphism
NGS: next-generation sequencing
CISeAL: Spanish acronym for the Center for Research on Health in Latin America
IGM: Spanish acronym for the Ecuadorian Military Geographic Institute
GPS: global positioning service
BLAST: Basic Local Alignment Search Tool
NCBI: National Center for Biotechnology Information
ANOVA: analysis of variance
NJ: neighbor-joining
SRA: sequence read archive database
